# Population structure and connectivity of the mountainous star coral, *Orbicella faveolata*, throughout the wider Caribbean region

**DOI:** 10.1002/ece3.3448

**Published:** 2017-10-03

**Authors:** John P. Rippe, Mikhail V. Matz, Elizabeth A. Green, Mónica Medina, Nida Z. Khawaja, Thanapat Pongwarin, Jorge H. Pinzón C., Karl D. Castillo, Sarah W. Davies

**Affiliations:** ^1^ Department of Marine Sciences University of North Carolina at Chapel Hill Chapel Hill NC USA; ^2^ Department of Integrative Biology University of Texas at Austin Austin TX USA; ^3^ Quantitative and Systems Biology University of California Merced CA USA; ^4^ Department of Biology Pennsylvania State University University Park PA USA; ^5^ Smithsonian Tropical Research Institute Smithsonian Institution Washington DC USA; ^6^ Department of Molecular Biology University of Texas Southwestern Medical Center Dallas TX USA; ^7^ Department of Biology Boston University Boston MA USA

**Keywords:** Caribbean, connectivity, coral reef, gene flow, population genetics

## Abstract

As coral reefs continue to decline worldwide, it becomes ever more necessary to understand the connectivity between coral populations to develop efficient management strategies facilitating survival and adaptation of coral reefs in the future. *Orbicella faveolata* is one of the most important reef‐building corals in the Caribbean and has recently experienced severe population reductions. Here, we utilize a panel of nine microsatellite loci to evaluate the genetic structure of *O. faveolata* and to infer connectivity across ten sites spanning the wider Caribbean region. Populations are generally well‐mixed throughout the basin (*F*_ST_ = 0.038), although notable patterns of substructure arise at local and regional scales. Eastern and western populations appear segregated with a genetic break around the Mona Passage in the north, as has been shown previously in other species; however, we find evidence for significant connectivity between Curaçao and Mexico, suggesting that the southern margin of this barrier is permeable to dispersal. Our results also identify a strong genetic break within the Mesoamerican Barrier Reef System associated with complex oceanographic patterns that promote larval retention in southern Belize. Additionally, the diverse genetic signature at Flower Garden Banks suggests its possible function as a downstream genetic sink. The findings reported here are relevant to the ongoing conservation efforts for this important and threatened species, and contribute to the growing understanding of large‐scale coral reef connectivity throughout the wider Caribbean.

## 1 INTRODUCTION

Over the past several decades, major physical, chemical, and ecological changes to the marine environment have compromised the health and persistence of coral reefs globally (Andersson & Gledhill, 2013; Hoegh‐Guldberg et al., 2007). In the wider Caribbean region specifically, large‐scale surveys of live coral cover estimate losses on the order of 60% since the 1980s (Jackson, Donovan, Cramer, & Lam, 2014). Particularly concerning is the disproportionate loss of critical framework species, such as the *Orbicella* species complex (*Orbicella annularis*,* Orbicella faveolata,* and *Orbicella franksi*), as their characteristic slow growth and long life spans prevent rapid reef recovery following mortality (Gladfelter, Monahan, & Gladfelter, 1978). *Orbicella* spp. previously functioned as dominant reef‐builders throughout the region; however, the abundance of this species complex has declined sharply (Bruckner & Bruckner, 2006; Edmunds, 2015), and it has largely been replaced by smaller “weedy” coral species with shorter generation times, such as *Agaricia* spp. and *Porites* spp. (Davies, Matz, & Vize, 2013; Green, Edmunds, & Carpenter, 2008; Knowlton, 2001). This transition has led to reduced structural habitat complexity and as a result, lower diversity and abundance of reef‐dependent fishes (Gratwicke & Speight, 2005; Risk, 1972) and invertebrates (Idjadi & Edmunds, 2006) on Caribbean reefs. In addition, it has been shown that declines of *Orbicella* spp. have been exacerbated by persistent recruitment failure (Hughes & Tanner, 2000; Van Woesik, Scott, & Aronson, 2014). These severe population declines coupled with a lack of recruitment led the National Marine Fisheries Service to classify the *Orbicella* species complex as threatened under the Endangered Species Act in 2014 (NMFS 2014).

Effective management of coral reefs relies on a sound understanding of the level to which spatially discrete populations are linked by dispersal. In its most basic sense, dispersal facilitates an influx of individuals to recipient reefs that can bolster population persistence demographically via immigration (Gaines, Gaylord, & Largier, 2003; Hastings & Botsford, 2003; Palumbi, 2003). Additionally, connectivity via larval dispersal also fosters the exchange of genetic variants, which acts to increase standing genetic variation (fuel for evolutionary adaptation) and facilitates the spread of advantageous alleles within and between reefs. In purple sea urchins (*Strongylocentrotus purpuratus*), for example, populations along the U.S. west coast were found to be genetically differentiated at certain loci due to adaptation to their local environment; yet, all alleles which confer an advantage in any one environment are spread at low frequencies almost everywhere else throughout the species range due to dispersal (Pespeni & Palumbi, 2013). As a result, connectivity primes populations to adapt to a range of environmental pressures across the extent of the species distribution. In this way, understanding the source–sink dynamics of larval supply and dispersal across a seascape provides insight into valuable reef areas for protection and can greatly enhance the effectiveness of marine conservation planning, especially in light of ongoing global change.

For corals and other sessile organisms, dispersal distance is primarily a function of pelagic larval duration (PLD), or the time required for a larva to become competent to settle (Selkoe & Toonen, 2011; Shanks, 2009). PLD varies across species, fertilization strategy, larval development rate, and responsiveness to settlement cues (Graham et al. 2008; Miller and Mundy 2003; Tebben et al. 2015; Underwood et al. 2009). *Orbicella faveolata* is a hermaphroditic broadcast spawner that releases its gametes synchronously following the full moon in the warmest month of the year (Szmant, 1991; Vize, 2006). *Orbicella* spp. larvae are generally competent to settle 5 days post‐fertilization (dpf; although see Davies, Meyer, Guermond, & Matz, 2014), and most will attempt to settle within 2 weeks if the appropriate cue is received (Szmant & Meadows, 2006; Wellington & Fitt, 2003). However, if the appropriate cue is unavailable, larvae of *Orbicella franksi* have been shown to successfully settle up to 120 dpf in the laboratory, highlighting the long‐distance dispersal potential of this species complex (Davies et al., 2017).

The once widely held paradigm that marine populations routinely exchange individuals across great distances has been challenged by mounting evidence that suggests local retention plays a much greater role in structuring populations at demographically relevant time scales than previously appreciated (Almany et al., 2007; Bode, Bode, & Armsworth, 2006; Cowen et al., 2000; Swearer et al., 2002). Over the last decade, the development of more sophisticated genetic analyses and biophysical models has allowed researchers to better predict where along the dispersal continuum from “open” to “closed” marine populations lie (Cowen & Sponaugle, 2009). To date, our understanding of *O. faveolata* population structure is limited to three regional studies within the Caribbean seascape, of which two observed genetic homogeneity between populations along the Florida Reef Tract (Baums, Johnson, Devlin‐Durante, & Miller, 2010) and between populations in Puerto Rico, the Yucatan, and lower Florida Keys (Severance & Karl, 2006). More recently, however, Porto‐Hannes et al. (2015) observed significant genetic structure within the Mesoamerican Barrier Reef System. These divergent findings illustrate that larval dispersal across the Caribbean may be influenced by multiple complex processes, which leads to uncertainty about the degree of population connectivity unless the entire basin is considered.

Here, we explore the patterns of *O. faveolata* population connectivity across the geographic extent of the wider Caribbean region using nine polymorphic microsatellite loci (Davies et al., 2013; Severance, Szmant, & Karl, 2004). In a study across a similar spatial scale, *O. annularis* (sister species to *O. faveolata*) was shown to differentiate into three genetic neighborhoods comprising the eastern, western, and central regions of the Caribbean (Foster et al., 2012). Using a combination of empirical genetic data with a biophysical projection model, Foster et al. (2012) refine the location of a previously described genetic break between populations in the eastern and western portions of the basin (Baums, Paris, & Cherubin, 2006; Vollmer & Palumbi, 2007) to the area between the British Virgin Islands to the east and Dominica to the west. It remains to be seen, however, whether *O. faveolata* follow the same patterns of genetic structure.

With the findings of this investigation, we specifically seek to achieve three primary goals: (i) quantifying the extent to which populations of *O. faveolata* are open or closed to long‐distance gene flow across the wider Caribbean, (ii) evaluating the uniformity of a previously established genetic break between eastern and western populations across taxa, and (iii) identifying unique patterns of substructure within the broader framework of *O. faveolata* population genetics in the region. This study contributes to a growing understanding of the dispersal capabilities of marine invertebrates in the wider Caribbean. In doing so, it helps to further inform regional conservation strategies for an endangered reef‐building coral and provides a foundation for future investigations into the mechanisms that underlie the observed patterns of population genetics.

## 2 MATERIALS AND METHODS

### 2.1 Sample collection

**Figure 1:**
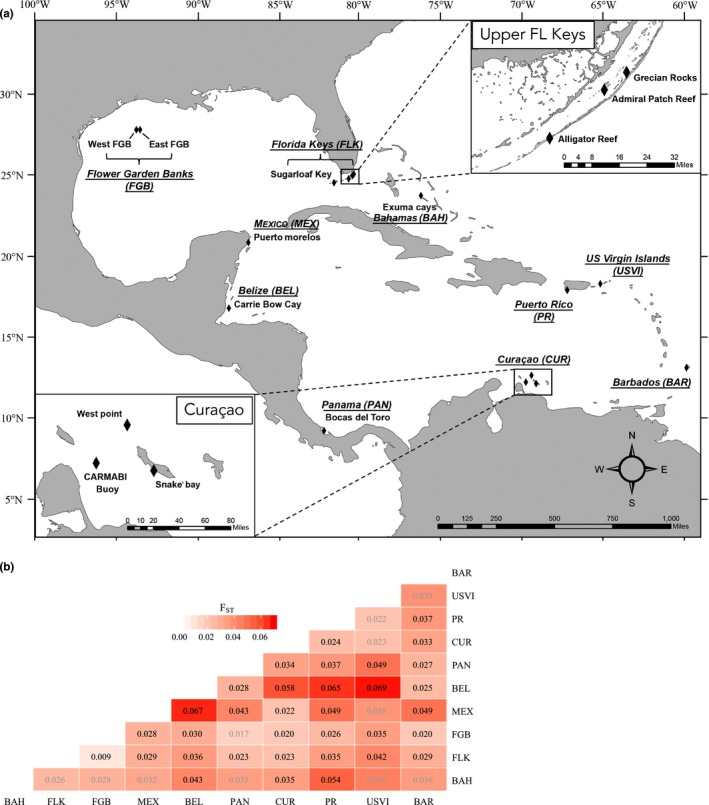
(a) Map of sampling sites. Individuals from neighboring reefs within each sampling site were grouped as single populations for all analyses performed. (b) Heat map of pairwise *F*_ST_ estimates between all sampling sites. Darker shades of red correspond to higher estimates of population differentiation via pairwise *F*_ST_. All bolded values are significant at the α = 0.05 level following sequential Bonferroni correction. Site abbreviations are as in Table 1 and are organized along the axes by relative geographic location in the basin

Between 1998 and 2015, a total of 416 tissue samples (~2 cm^2^) of the mountainous star coral, Orbicella faveolata, were collected from 10 sites that span the extent of the wider Caribbean seascape (Figure 1a, Table 1). Specifically, samples from the Bahamas and Upper Florida Keys (excluding Sugarloaf Key) were collected between 1998 and 2004, and all others were collected between 2012 and 2015. At several locations, samples were collected from a group of neighboring reefs. To account for the large spatial scale of interest in this study, these samples were pooled as single populations for all analyses performed (Table 1; see Appendix S1 for further justification regarding pooling). All samples were collected between 5 and 15 m depth with the exception of those from the Flower Garden Banks (FGB), which were collected at 20–25 m depth.

**Table 1:** Sampling sites and estimates of genetic diversity

Sampling site	Reef locations	Lat	Long	*N*	*N*_*A*_	*N*_PA_	AR	*H*_*O*_	*H*_*E*_	*E*
Bahamas (BAH)	*Exuma Cays*	23.783	−76.118	11 (1)	6.67	0	6.380	0.505	0.633	0.532
Barbados (BAR)		13.100	−59.634	25 (1)	9.00	0.78	6.623	0.419	0.665	0.598
Belize (BEL)	*Carrie Bow Cay*	16.802	−88.077	35 (1)	11.56	1.56	6.940	0.491	0.660	0.338
Curacao (CUR)	*Snake Bay*	12.125	−68.973	46 (8)	10.33	0.78	6.175	0.551	0.647	0.518
	*West Point*	12.637	−69.270							
	*CARMABI Buoy*	12.210	−69.619							
Flower Garden Banks (FGB)	*East*	27.900	−93.583	90 (1)	14.00	1.89	6.932	0.486	0.694	0.746
	*West*	27.883	−93.817							
Florida Keys (FLK)	*Alligator Reef*	24.842	−80.624	54 (13)	10.56	1.22	6.390	0.538	0.689	0.568
	*Grecian Rocks*	25.119	−80.302							
	*Admiral Patch*	25.045	−80.395							
	*Sugarloaf Key*	24.591	−81.536							
Mexico (MEX)	*Puerto Morelos*	20.877	−86.858	28 (11)	7.67	0.22	5.433	0.476	0.620	0.312
Panama (PAN)	*Bocas del Toro*	9.200	−82.150	21 (1)	8.89	0.33	6.636	0.582	0.687	0.531
Puerto Rico (PR)	*Media Luna*	17.935	−67.034	48 (1)	9.22	0.44	5.943	0.392	0.608	0.343
	*Turromote*	17.935	−67.019							
US Virgin Islands (USVI)	*Flat Cay West*	18.318	−64.991	11 (1)	6.22	0.22	6.008	0.461	0.582	0.447
	*Botany Bay*	18.358	−65.033							

*N*: number of unique multilocus genets (number of clonal individuals listed in parentheses); *N*_*A*_: number of alleles; *N*_PA_: number of private alleles; AR: allelic richness, calculated as the population mean of locus‐specific allelic richness; *H*_*O*_: observed heterozygosity; *H*_*E*_: expected heterozygosity; *E*: Simpson's evenness index

At all locations except the lower Florida Keys (Sugarloaf Key), fragments of tissue were excised from adult colonies using a hammer and chisel and were placed in individual plastic bags filled with seawater. Tissue samples collected at Sugarloaf Key were siphoned from the coral surface using a plastic syringe to minimize physical damage, in compliance with permit requirements. The filter paper containing each tissue sample was placed in a labeled plastic bag filled with a 96% ethanol (EtOH) solution. At all sites, colonies of *O. faveolata* were identified visually based on the morphological criteria outlined in Weil and Knowton (1994). Care was taken to avoid sampling connected colonies and those within 5 m of each other in order to minimize the possibility of sampling clones.

All samples were then transported in an insulated cooler to the laboratory for preservation. Samples from the Bahamas, Barbados, Belize, Florida Keys, and USVI were transferred to DMSO buffer (20% dimethyl sulfoxide and 0.25 ethylenediaminetetraacetic acid (EDTA) in NaCl‐saturated water), while those from the FGB were transferred to 96% EtOH. All of the above were stored at −20°C. Samples from Curaçao, Mexico, Panama, and Puerto Rico were flash‐frozen in liquid nitrogen and stored at −80°C.

Note, to maintain consistent terminology throughout the manuscript, the terms *sampling site*,* site* and *population* will be used interchangeably to refer to the 10 sampling sites discussed above and outlined in Table 1, while the term *region* will refer to four specific groups of these sampling sites that are identified statistically, as described below and in Table 2.

**Table 2:** Analysis of molecular variance (AMOVA) among and within sampling sites and regions

Model	Source of variation	*df*	Sum of squares	Variance components	Variation (%)	Fixation indices
Four regions (PCoA‐derived)	Among regions	3	60.122	0.058	1.83	*F*_CT_ = 0.018*
	Among sampling sites within regions	6	55.196	0.093	2.91	*F*_SC_ = 0.030*
	Among individuals within sampling sites	728	2214.052	3.041	95.26	*F*_ST_ = 0.047*
	Total	737	2329.37	3.193	100	
10 sampling sites	Among sampling sites	9	115.318	0.138	4.35	
	Among individuals within sampling sites	728	2214.052	3.041	95.65	*F*_ST_ = 0.043*
	Total	737	2329.37	3.179	100	

The four regions identified by principal coordinate analysis include (1) PR, USVI, CUR, and MEX, (2) FGB, FLK, and BAH, (3) BAR and PAN, and (4) BEL. Fixation indices denote the variation among regions (*F*_CT_), among sampling sites within each region (*F*_SC_) and among individuals within sampling sites (*F*_ST_). An * denotes significance at the α = 0.05 level based on 9999 random permutations

### 2.2 DNA extraction and sequencing

In preparation for DNA isolation, tissue was removed from storage solution, submerged in digest buffer (100 mmol/L NaCl, 10 mmol/L Tris‐Cl pH 8.0, 25 mmol/L EDTA pH 8.0, 0.5% SDS, 0.1 mg/mL Proteinase K, and 1 μg/mL RNaseA) and was heated to 42°C for 1 hr. DNA was then isolated following a standard phenol–chloroform extraction protocol (Davies et al., 2013). A panel of nine microsatellite loci was amplified using 10 μL multiplex polymerase chain reactions following Davies et al. (2013). PCR products were visualized on agarose gels to verify amplification, and molecular weights were assessed using an ABI 3130XL capillary sequencer with an ROX‐labeled size standard. Alleles were scored, and genotypes were assigned based on amplicon size using GeneMarker 1.70. If loci amplifications of an individual failed within multiplex reactions, additional PCRs were attempted to assemble the most complete dataset possible.

### 2.3 Statistical analysis

Before further analysis, the probability of identity (*P*_ID_), or the probability that two independently sampled individuals within a population share an identical multilocus genotype by chance, was calculated using GenAlEx v6.502 (Peakall & Smouse, 2006) and was found to be ≤2.6E−08 for all populations. Hence, any identical genotypes found in the dataset were presumed to be clones and were removed (*n* = 39). Clone mates were only found within the same geographic site. Additionally, samples with data missing for more than three loci were removed, leaving 369 unique genets for downstream analysis.

FSTAT v2.9.3 (Goudet, 1995, 2001) was used to calculate allelic richness and to test for linkage disequilibrium (LD) between all pairs of loci in each sampled population (7,200 permutations). Allelic richness is a particularly robust measure of gene diversity, as it incorporates a rarefaction procedure to standardize estimates to the smallest common sample size (*n* = 11). Genepop v4.5.1 (Raymond & Rousset, 1995; Rousset, 2008) was used to calculate observed and expected proportions of heterozygotes and to perform single‐locus tests of conformity to Hardy–Weinberg (HW) equilibrium using Fisher's exact test based on Markov chain iterations (10,000 dememorization steps, 500 batches of 10,000 iterations). To address deviations from HW proportions, the frequency of null alleles was estimated in FreeNA (Chapuis & Estoup, 2007) using the EM algorithm of Dempster, Laird, and Rubin (1977). Additionally, to determine whether populations differ significantly in allelic richness and heterozygosity, a randomized block ANOVA and Tukey's honest significant difference (HSD) test were performed in R using the sampling sites as treatment levels and locus as a covariate.

Global *F*_ST_ was calculated in FSTAT v2.9.3 with 95% confidence intervals constructed by bootstrapping over all loci. To investigate the hierarchical partitioning of diversity within and among sampling sites and regions, an analysis of molecular variance (AMOVA) procedure was implemented in GenAlEx v6.502 using an infinite allele model and 9999 random permutations. The demarcation of four discrete regions across the sampling range was guided by the apparent clustering of populations based on pairwise estimates of differentiation, which were also calculated via AMOVA. Pairwise *F*_ST_ was calculated to enable comparisons to past research; however, Hedrick's (2005) $G_{\mathrm{ST}}^{\prime \prime }$ statistic is also reported, as it incorporates standardized estimates for total and within‐population expected heterozygosity developed by Nei and Chesser (1983) in order to correct for small sample sizes, as well as an adjustment when a small number of populations are sampled (Meirmans & Hedrick, 2011). Thus, while the overall patterns remain largely unchanged between the two estimates, given the sampling scheme in this study, pairwise $G_{\mathrm{ST}}^{\prime \prime }$ values more accurately represent genetic differentiation and were therefore used to visualize population structure via principal coordinates analysis (PCoA) and to test for the presence of isolation by distance. Both estimates were calculated using GenAlEx v6.502.

Isolation by distance was assessed by calculating the correlation between $G_{\mathrm{ST}}^{\prime \prime }/\left(1-G_{\mathrm{ST}}^{\prime \prime }\right)$ and the natural logarithm of over‐water distance using a Mantel test in *R* (9999 permutations), as recommended by Rousset (1997). Over‐water distance was approximated in Google Earth as the shortest path between two sites without crossing land.

A Bayesian model‐based clustering method was used to infer the number of genetically distinct populations and assign individuals proportionally to inferred populations. This analysis was implemented in STRUCTURE v2.3.4 (Falush, Stephens, & Pritchard, 2003, 2007; Pritchard, Stephens, & Donnelly, 2000). Ten independent replicate runs were performed for each *K* from 1 to 10 using a burn‐in period of 300,000 followed by 1,000,000 MCMC repetitions, where *K* represents the number of genetic populations. Mean membership coefficients (*q*) are calculated for each individual, which describe the likelihood that an individual belongs to each of the inferred populations. The clustering algorithm was assisted using the 10 sampling sites as a prior (Hubisz, Falush, Stephens, & Pritchard, 2009), and allele frequencies were assumed to be correlated between populations. The most likely number of genetic clusters was then determined using the ad hoc statistic Δ*K* based on the posterior probability of the data following the methods of Evanno, Regnaut, and Goudet (2005), as implemented in STRUCTURE Harvester (Earl & vonHoldt, 2012). Simulation results were compiled in CLUMPP v1.1.2 (Jakobsson & Rosenberg, 2007) and visualized using distruct v1.1 (Rosenberg, 2004).

Lastly, Simpson's index (*E*) was calculated for each sampling site to describe qualitatively the evenness of representation from each of the inferred genetic clusters:

$$E=\;\frac{D}{D_{\max}}\;=\;\frac{1/\sum_{i=1}^{n}p_{i}^{2}}{n}$$

where *D* represents Simpson's diversity index, *p*_*i*_ represents the mean probability of assignment to each inferred cluster (1–5), and *n* is the total number of clusters. This index ranges from 1/*D*_max_ (in this case, 0.20) to 1, which would indicate an equal proportion of all clusters.

## 3 RESULTS

### 3.1 Genetic diversity

Genetic diversity as measured by expected heterozygosity and mean allelic richness were moderately high in all populations, with values ranging from 0.582 to 0.694 and from 5.433 to 6.940, respectively (Table 1). Allelic richness was greatest in Belize and the Flower Garden Banks (FGB) and lowest in Mexico, although none of the differences between populations were statistically significant (randomized block ANOVA, *p *=* *.113). Similarly, while ANOVA results provide evidence that heterozygosity may differ between populations (randomized block ANOVA, *p *=* *.025), the more statistically stringent Tukey's HSD test revealed no significant pairwise differences between populations. Thus, there is no sufficient evidence to conclude that heterozygosity in any population was greater or less than that of any other.

Twenty‐two of 90 locus‐by‐population tests revealed evidence of heterozygote deficiency after sequential Bonferroni correction, of which 18 were associated with four particular microsatellite loci: Mfav5_CGA, Mfav4_TTTG, Mfav6_CA, and Mfav8_CAA (Table S1). This pattern of locus‐specific heterozygote deficiency is often interpreted as a symptom of null alleles, and in many studies, it has been used as grounds for removing affected loci from analysis. However, we find the frequency and influence of null alleles in our dataset to be low and insignificant (max $\hat{\gamma }$ = 0.22; Table S2), suggesting that the observed heterozygote deficit may instead reflect underlying biological patterns. Hence, all loci were retained for subsequent analysis. Refer to the supplemental information for greater detail regarding our treatment and assessment of null alleles for this dataset.

Significant linkage disequilibrium (LD) was observed in ~10% (33 of 360) of locus pair comparisons after sequential Bonferroni adjustment. No locus pairs were significantly associated across more than three sampling sites, suggesting that the observed disequilibrium is likely not a result of physical linkage of the markers (Table S3). Interestingly, 31 of the 33 significant tests of association occurred in samples from Florida, Mexico, and Curaçao, which also accounted for ~82% (32 of 39) of the clonal individuals removed from the dataset prior to analysis (Table S4).

### 3.2 Population structure via *F*‐statistics

Global *F*_ST_ was low but significantly different from zero (*F*_ST_ = 0.038; 95% confidence interval: 0.024 to 0.061). Pairwise *F*_ST_ and $G_{\mathrm{ST}}^{\prime \prime }$ estimates between localities ranged from 0.009 to 0.065 and 0.013 to 0.290, respectively (Figure 1b, Table S5). Only seven of 17 comparisons involving the Bahamas or US Virgin Islands were statistically significant after sequential Bonferroni correction, likely due to the small sample sizes from these sites (*n* = 11 at each location); however, all other comparisons except Panama‐FGB were significant.

**Figure 2:**
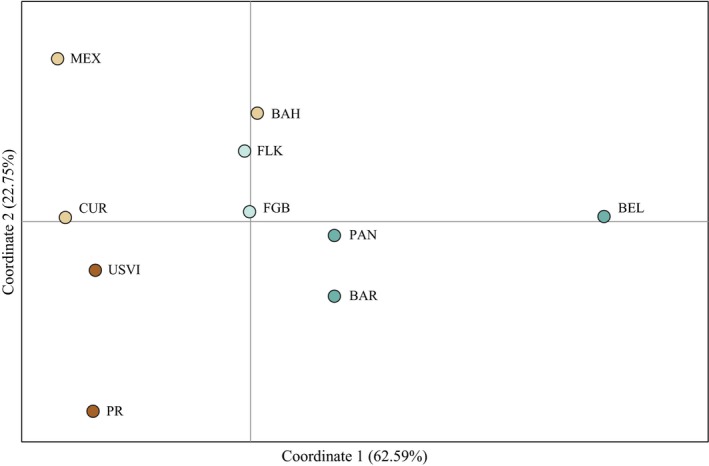
Principal coordinates analysis (PCoA) via pairwise estimates of $G_{\mathrm{ST}}^{\prime \prime }$. The distance separating points in ordinate space along each axis corresponds to genetic divergence as estimated via pairwise $G_{\mathrm{ST}}^{\prime \prime }$. Percentages indicate the proportion of variation in the data explained by each axis. Marker colors denote the dominant genetic cluster within each population as inferred by STRUCTURE analysis. Site abbreviations are as in Table1

A principal coordinates analysis based on pairwise $G_{\mathrm{ST}}^{\prime \prime }$ values reveals four distinct clusters segregated along principal coordinate (PC) 1, which explains 62.59% of the variation in these data (Figure 2). Most notably, the population in Carrie Bow Cay, Belize, was distantly isolated from all other populations and was most highly differentiated from its closest geographic neighbor in Puerto Morelos, Mexico. The US Virgin Islands, Puerto Rico, Curaçao, and Mexico group along PC1, but diverge considerably along PC2, which explains 22.75% of variation. The remaining groupings are composed of the Bahamas, Florida Keys and the Flower Garden Banks, and Barbados and Panama, respectively. An AMOVA detected low, but significant differentiation between these four regions and among populations within these regions, explaining 1.83% and 2.91% of the total variance in allelic frequencies, respectively (*p *<* *.001; Table 2).

**Figure 3:**
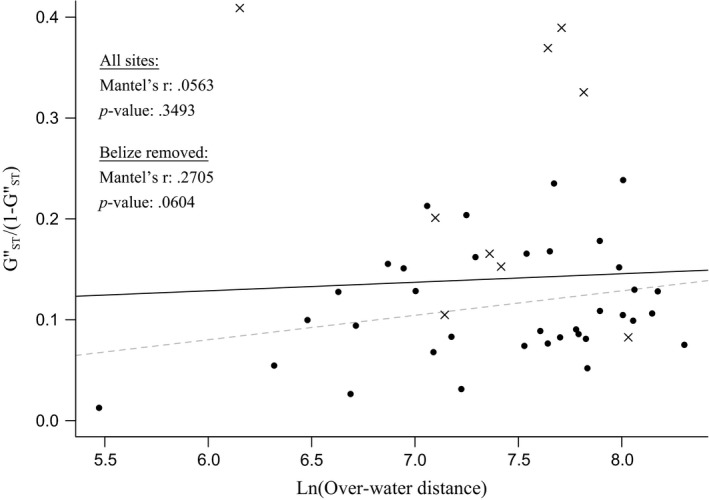
Isolation by distance observed for *Orbicella faveolata*. Pairwise genetic differentiation [$G_{\mathrm{ST}}^{\prime \prime }/\left(1-G_{\mathrm{ST}}^{\prime \prime }\right)$] as a function of the natural logarithm of over‐water distance between populations. Correlations are plotted for pairwise estimates between all populations (solid line) and excluding Belize (dashed line). × indicates a pairwise estimate involving Belize

Overall, we found no evidence that genetic differentiation between sites [$G_{\mathrm{ST}}^{\prime \prime }/\left(1-G_{\mathrm{ST}}^{\prime \prime }\right)$] was correlated with pairwise over‐water distance (Mantel's *r* = 0.0563, *p *=* *.3493; Figure 3). However, if we remove Belize from analysis as an outlier based on observed genetic isolation, a positive trend emerges that approaches significance at the α = 0.05 level (Mantel's *r* = 0.2705, *p *=* *.0604; Figure 3). This suggests that geographic distance is not the principal structuring force across the study range, but may be a weak, underlying mechanism of genetic isolation existing within a complex system of oceanographic and biological factors.

### 3.3 Population structure via clustering analysis

**Figure 4:**
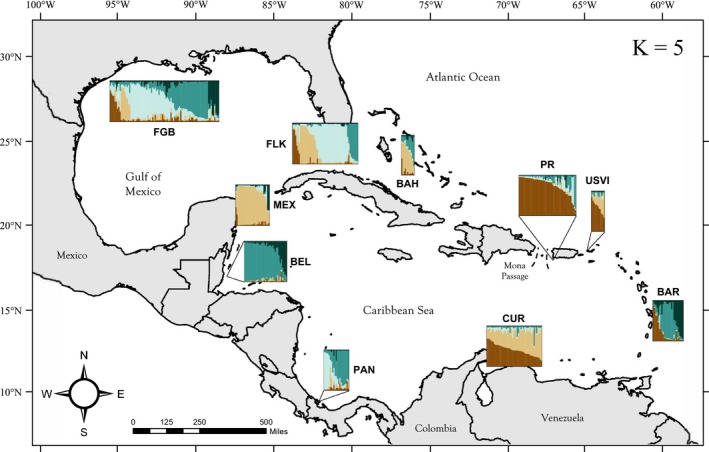
STRUCTURE population assignments for *Orbicella faveolata* across the wider Caribbean region. Thin vertical columns in each population block represent individual samples and their associated probability of assignment to *K* = 5 color‐coded genetic clusters (Brown, Tan, Light Blue, Teal, and Dark Blue). Individual columns are of uniform width; thus, the size of population blocks is proportional to the number of samples representing each population. Site abbreviations are as in Table1

Based on the ad hoc statistic Δ*K* (Evanno et al., 2005), the optimal solution for STRUCTURE analysis exhibited five distinct genetic clusters throughout the sampling range (Brown, Tan, Light Blue, Teal, Dark Blue colors in Figure 4; Figure S2, S3). All except the Dark Blue cluster were dominant (>50%) in at least one of the sampling sites; however, only three of the 10 populations (Belize, Mexico and Puerto Rico) could be assigned unambiguously to one cluster. Rather, the inferred pattern of population structure reflected considerable mixing across the seascape consistent with the low global *F*_ST_ observed. The FGB, in particular, exhibited a markedly even distribution of individuals assigning to each of the five genetic clusters. Of the 10 sampling sites, Simpson's evenness was highest in the FGB (*E *=* *0.746) and lowest in Mexico, Belize, and Puerto Rico (*E *=* *0.312, 0.338 and 0.343, respectively). All other populations exhibited intermediate values within this range (Table 1).

Basin‐wide patterns of genetic differentiation based on STRUCTURE results generally mirrored those revealed by pairwise estimates of *F*_ST_ and $G_{\mathrm{ST}}^{\prime \prime }$. Specifically, three main observations were revealed:

1. The Brown cluster occurred predominantly and almost exclusively in the eastern Caribbean populations, with the exception of Barbados.
2. The FGB and Florida Keys populations seemed to reflect a similar mixture of individuals of the Tan, Light Blue, and Teal clusters. In particular, the Light Blue cluster was restricted primarily to these two populations.
3. Despite a physical separation of only 470 km, populations from Belize and Mexico shared virtually no similarity in their clustering assignments. Each was >75% associated with the Teal (Belize) and Tan (Mexico) cluster with <3% of each population associating with the dominant cluster of the other population, highlighting significant differentiation across these two populations.

## 4 DISCUSSION

### 4.1 East–West genetic barrier

Overall, our results illustrate that populations of *Orbicella faveolata* are relatively well‐mixed across the wider Caribbean (*F*_ST_ = 0.038), although within this broad setting, we find complex patterns of substructure arising at both local and regional scales. Our analysis indicates that eastern populations diverge considerably from populations to the west, which reflects patterns of connectivity previously described for *Acropora palmata* (Baums, Miller, & Hellberg, 2005; Baums et al., 2006), *Acropora cervicornis* (Galindo, Olson, & Palumbi, 2006), and *Orbicella* (previously: *Montastraea*) *annularis* (Foster et al., 2012). Notably, this finding contrasts with that of Severance and Karl (2006), who found significant divergence between populations of *O. annularis* in Puerto Rico, Mexico, and Florida, but not between populations of *O. faveolata*.

Moreover, our clustering results reveal a significant level of gene flow between Curaçao and Mexico, suggesting that the southern extent of this genetic break may be more pervious to dispersal than previously acknowledged (Baums et al., 2006; Foster et al., 2012). Oceanographic models demonstrate that westward larval dispersal from Puerto Rico in the north is physically impeded by complex oceanographic patterns associated with the Mona Passage (Baums et al., 2006; Cowen, Paris, & Srinivasan, 2006), but the mechanisms that would constitute a barrier at the southern extent of the basin are not as clear. Throughout the seascape, surface transport is dominated by the strong flow of the Caribbean Current, which enters the basin through the southern Lesser Antilles in the east and travels west‐northwest toward the Yucatan Peninsula (Alvera‐Azcárate, Barth, & Weisberg, 2009; Richardson, 2005). The swiftest portion of this current creates a nearly direct corridor along the Venezuelan coast that passes near Curaçao en route to Mexico, reaching mean velocities of 80–120 cm/s (Richardson, 2005). With larval competency lasting up to 30 days, as has been shown for *O. faveolata* (Szmant & Miller, 2006), these current speeds are more than sufficient to maintain significant connectivity between Curaçao and Mexico, particularly on the evolutionary timescale addressed by *F*_ST_ and STRUCTURE analyses.

We also find that Barbados does not conform to a simple east–west dichotomy. Rather, it exhibits greater genetic similarity to populations in the western Caribbean than to nearby localities in the east. A similar pattern of local segregation has been seen previously in soft corals (Andras, Rypien, & Harvell, 2013) and reef fish (Paris & Cowen, 2004) and has been attributed primarily to the local flow environment. Larvae of the bicolor damselfish, *Stegastes partitus*, exhibit unusually high retention rates around Barbados due to their ability to migrate vertically within the water column and take advantage of a persistent onshore current at depth (Cowen & Castro, 1994; Paris & Cowen, 2004; Paris et al., 2002). While the poor swimming proficiency of most marine invertebrate larvae likely limits their ability to actively adjust their position in the water column to the same extent as reef fish, we suggest that a similar oceanographic mechanism may be at work to locally retain larvae of *O. faveolata* in Barbados. Yet, while this may help explain the observed genetic isolation from nearby localities in the eastern Caribbean, the apparent connectivity between Barbados and western sites, such as Panama, Belize and the FGB, suggests that the processes governing larval dispersal from Barbados are complex and likely function at multiple spatial and temporal scales.

### 4.2 Evidence of larval retention in southern Mesoamerican Barrier Reef

Perhaps our most striking finding is the relatively strong population differentiation between Carrie Bow Cay, Belize and Puerto Morelos, Mexico (*F*_ST_ = 0.063), two sites within the Mesoamerican Barrier Reef System (MBRS) separated by only 470 km. With the use of five microsatellites, Porto‐Hannes et al. (2015) did not detect significant differentiation between *O. faveolata* populations near these two sites. However, we were able to distinguish significant structure at this small spatial scale, likely due to the additional four microsatellite markers utilized.

We hypothesize that certain features of the prevailing oceanographic patterns in this area may underlie the strong local differentiation observed here as well as the overall genetic isolation of the Belize population from the rest of the wider Caribbean. Notably, the MBRS is characterized by two divergent flow regimes originating where the Caribbean Current encounters the Yucatan Peninsula (Tang, Sheng, Hatcher, & Sale, 2006). To the north, the Caribbean Current veers into the Yucatan Channel maintaining high northward current velocities as it travels into the Gulf of Mexico. Contrastingly, the southern extent of the MBRS is characterized by the preponderance of submesoscale eddies (~10–100 km), which are shed from the Caribbean Current and comprise a persistent cyclonic gyre covering much of the Gulf of Honduras (Carrillo et al., 2015; Lindo‐Atichati, Curcic, Paris, & Buston, 2016; Paris, Chérubin, & Cowen, 2007; Tang et al., 2006). Using a nested grid ocean circulation model, Tang et al. (2006) illustrate that during the month of August, when *O. faveolata* are known to spawn, this recirculation cell severely constrains the dispersal of simulated larvae released from Glovers Reef Atoll (just offshore from Carrie Bow Cay) to inshore regions of the southern Belize shelf. This provides compelling support for a genetic break between Carrie Bow Cay and Puerto Morelos. However, further research is needed to determine whether such a barrier to dispersal could persist over the timescale required to generate the level of population divergence observed here.

### 4.3 Downstream populations as genetic sinks

A strong signal of connectivity between populations in Mexico, the Florida Keys, and the Bahamas is consistent with previous findings of virtually no genetic structure across this range in *A. palmata* (Baums et al., 2005) and *O. faveolata* (Severance & Karl, 2006). Interestingly, Severance and Karl (2006) find that this pattern contrasts with that of *O. annularis*, a sister species within the *Orbicella* complex, which exhibits significant population divergence across the same range (rho = 0.02–0.23). The authors propose that such a stark difference between two closely related taxa may have arisen through the accumulation of chance differences in recruitment success across years. Additionally, such contrasting patterns could result from genetic introgression or the unintended inclusion of sibling species in their analysis. To be sure, future work is needed to elucidate these species‐specific dynamics.

A particularly unique aspect of the present study is the incorporation of the FGB in our analysis of Caribbean‐wide population genetics. Located in the northwestern reaches of the Gulf of Mexico, the FGB is distantly separated from other reefs in the basin and is only occasionally accessible to dispersing larvae via northbound eddies shed from the loop current (Lugo‐Fernández et al., 2001;). Yet, despite its remote location, we find that its genetic signature represents a nearly even mixture of genets originating from populations across the whole Caribbean basin, a pattern that can be characteristic of a genetic sink (Andras et al., 2013). Additionally, very low population differentiation between FGB and the Florida Keys (*F*_ST_ = 0.009) suggests relatively strong connectivity between these two sites. The net direction of larval transport between the sites remains unknown; however, a recently developed biophysical model demonstrates a mechanism by which larvae of *O. franksi* are able to disperse from FGB to reefs in the Florida Keys and Bahamas (Davies et al., 2017). Thus, while the FGB may be interpreted as a possible genetic sink relative to populations across the Caribbean basin, it may also provide larval subsidy to reef areas that are downstream of the prevailing Loop and Caribbean Currents. It is worth pointing out that the reefs of the FGB remain among the healthiest in the wider Caribbean (Aronson, Precht, Murdoch, & Robbart, 2005; Zimmer et al., 2010) and stand in stark contrast to the deteriorating reefs of the Florida Keys (Ruzicka et al. 2013). Significant gene flow between these two populations may become crucial to the long‐term recovery of the Florida Keys Reef Tract.

### 4.4 Heterozygote deficiency putatively due to introgressive hybridization

The discovery of locus‐specific heterozygote deficiency in our dataset is not uncommon among studies of marine populations and is most often attributed to some combination of the following factors: the occurrence of null alleles, the Wahlund effect, inbreeding, and/or selection against heterozygotes (sexual or otherwise; Allendorf et al. 2013). The observed deficiency cannot be attributed exclusively to any one factor with certainty; however, several can be ruled out for this study. For instance, we show that the estimated frequency of null alleles within this dataset is relatively low and that their effect on patterns of population differentiation is negligible. Additionally, the Wahlund effect describes the reduction in heterozygosity that is observed when multiple genetically differentiated populations are inadvertently collected as one sample (Dharmarajan, Beatty, & Rhodes, 2013; Wahlund, 1928). This is expected to occur when there are a number of highly differentiated populations; yet, given the overall genetic similarity of the individuals in this study (*F*_ST_ = 0.038), a significant Wahlund effect is relatively unlikely. Furthermore, while it is possible that inbreeding may play a role, it would be expected to affect all loci relatively equally (Gaffney, Scott, Koehn, & Diehl, 1990; Slate et al., 2004; Waples, 2014), which is not seen in the sampled populations studied here.

Rather, in this case, we hypothesize that the observed disagreement with Hardy–Weinberg expectations is most likely due to genetic introgression and/or the inadvertent sampling of sibling species within the *Orbicella* complex. All but one of the microsatellite markers used in this study (Mfav8_CAA) have been shown to amplify in other *Orbicella* species with ≥80% success (Davies et al., 2013; Severance et al., 2004). Additionally, the three *Orbicella* species occur in sympatry throughout their range with overlapping distributions across depth and have shown a capacity for interspecific hybridization, particularly near the northern extent of their distribution (Budd & Pandolfi, 2004; Fukami et al., 2004; Knowlton et al., 1997; Szmant, Weil, Miller, & Colon, 1997). Thus, while great care was taken to identify and sample colonies of *O. faveolata*, it can be quite difficult in some locations to visually distinguish this species from its close relatives in the *Orbicella* complex and especially from hybridized “intermediate” morphologies, as has been documented in the Florida Keys and Bahamas (Budd & Pandolfi, 2004; Fukami et al., 2004; Manica & Carter, 2000; Szmant et al., 1997). A deficit in heterozygotes will arise when two differentiated populations (or in this case, species) interbreed to some extent, but mate preferentially and with greater success within their own population (Roques, Sévigny, & Bernatchez, 2001). This has been shown to occur within the *Orbicella* species complex, whereby interspecific fertilization was achieved in experimental crosses between *O. faveolata* and its sibling species, but at rates significantly lower than what is observed between conspecifics (Szmant et al., 1997).

Introgressive hybridization may also account for the significant tests of linkage disequilibrium, which are seen predominantly in Florida, Mexico, and Curaçao, through the introduction of foreign alleles into the sampled gene pools. Even without strong linkage between alleles within their parent population, the joint introduction of novel alleles into a hybrid would imitate linkage to an investigator. It is important to note that the association between loosely linked loci would decay rapidly at a rate equal to the recombination rate and therefore must be a result of hybridization within the last few generations (Goodman, Barton, Swanson, Abernethy, & Pemberton, 1999). However, it is possible that this signal of linkage disequilibrium would persist through time if *F*_1_ and *F*_2_ hybrids are less fit than the parental types, either in viability or fecundity. Such a scenario, described by Barton and Hewitt (1985) as the Tension Zone model, would maintain a stable, but marginal population of first‐ or second‐generation hybrids.

Interestingly, the same populations with the highest observed linkage disequilibrium (Florida Keys, Mexico, and Curaçao) also account for the large majority (~82%) of clonal individuals removed from the dataset prior to analysis. This uneven geographic distribution of multilocus clones may reflect underlying ecological circumstances at these locations. However, to confirm the true extent and dynamics of hybridization and a possible association with asexual reproduction, a more comprehensive investigation of allele frequencies within these populations that includes all possible parental genotypes is required.

## 5 CONCLUSION

The results of this study advance the current understanding of population connectivity throughout the wider Caribbean and offer insight into local and regional patterns of genetic structure for the important reef‐building coral *O. faveolata*. Within the context of strong overall connectivity across the seascape, we find further support for a genetic break between eastern and western populations as has been observed for other coral species. We discover evidence for strong differentiation between neighboring populations within the MBRS and also find evidence that the FGB may function as a genetic sink for the Caribbean basin.

From a management perspective, it is essential to bear in mind that the findings and interpretations presented here describe patterns of population connectivity that have arisen over an evolutionary timescale (thousands of generations). Accordingly, we can use these data to infer the extent to which genetic material is shared between populations, which can have important implications for overall genetic variation and potential for adaptation. However, estimates of genetic divergence alone provide little information on demographic connectivity, that is, whether or not the per‐generation rate of migration between populations is large enough to significantly affect population growth. Understanding both the genetic and demographic connectivity among populations is critical to the management of *O. faveolata*, and we therefore encourage the use of the genetic patterns discussed here to inform future investigations into the magnitude of larval dispersal throughout the wider Caribbean seascape.

## AUTHOR CONTRIBUTIONS

SWD, MVM, and MM conceived and designed the study and sampling scheme. SWD, MVM, EAG, MM, NZK, TP, and JHPC acquired permits, collected coral tissue samples, or isolated DNA. SWD, NZK, and TP completed all microsatellite multiplex assays. JPR analyzed the microsatellite data. All authors contributed to writing of the manuscript. The authors thank Carly Kenkel, Marissa Nuttall, Eli Meyer, Bill Fitt, Dan Thornhill and Scott Santos for their contribution to sample collection and preservation. This research was supported by the National Science Foundation (DEB‐1054766, OCE‐1642311, OCE‐1442206, OCE‐9906976 and OCE‐0137007) and by the NOAA National Undersea Research Program through both the Caribbean Marine Research Center on Lee Stocking Island, Bahamas and the Florida Keys Dayboat Program run by the University of North Carolina at Wilmington. We also acknowledge all permitting authorities for previously assembled coral collections.

## DATA ACCESSIBILITY

Microsatellite genotype data and STRUCTURE output files are made available via the DRYAD Digital Repository; https://doi.org/10.5061/dryad.15qf2.
