# Supplementary figures and images for: Population structure and connectivity of the mountainous star coral, Orbicella faveolata, throughout the wider Caribbean region
Source: Ecol Evol. 2017 Oct 3;7(22):9234–46. doi: 10.1002/ece3.3448 (PMC5696396; doi:10.1002/ece3.3448)

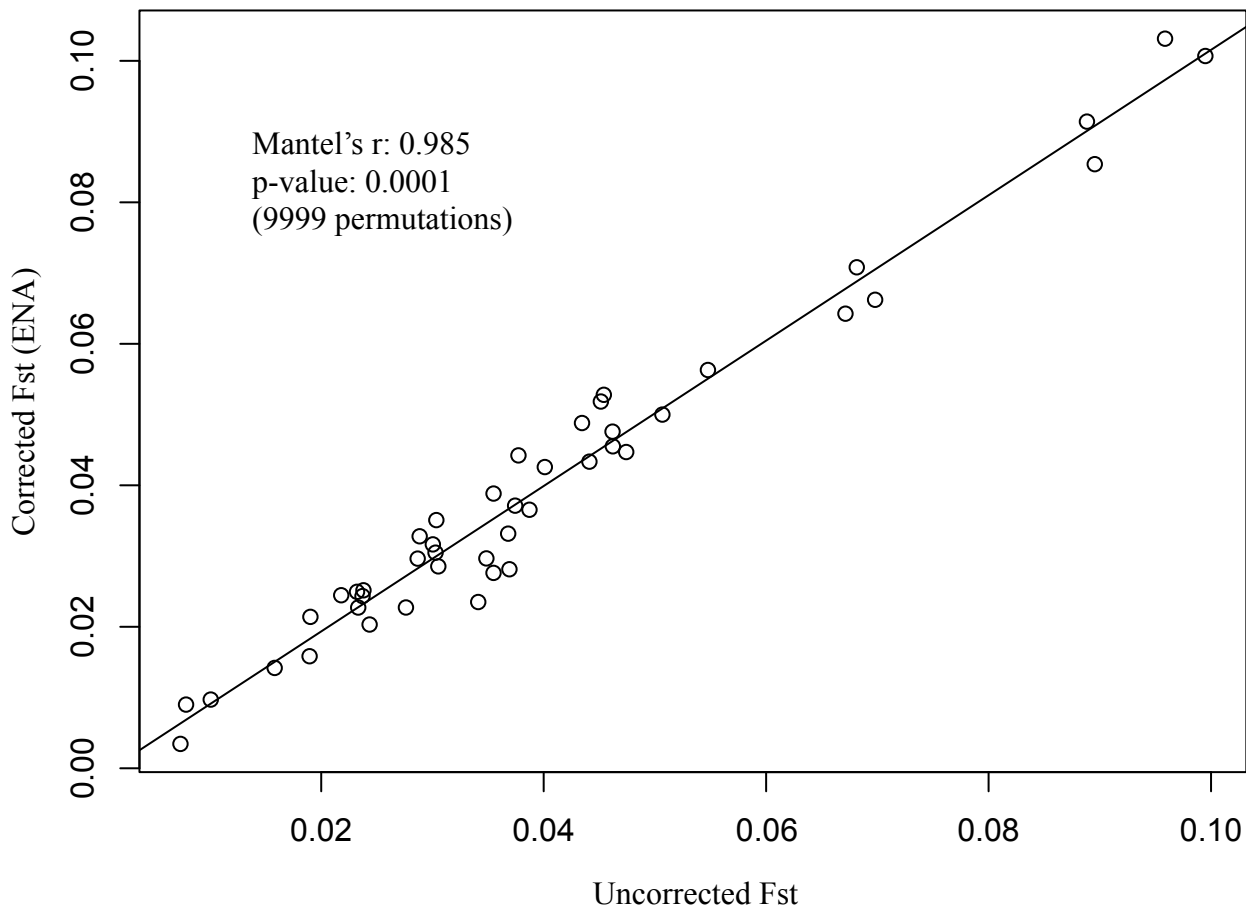

Supplement: Supplementary file 1 [file ECE3-7-9234-s001.pdf]

$$\text{DeltaK} = \text{mean}(|L''(K)|) / \text{sd}(L(K))$$

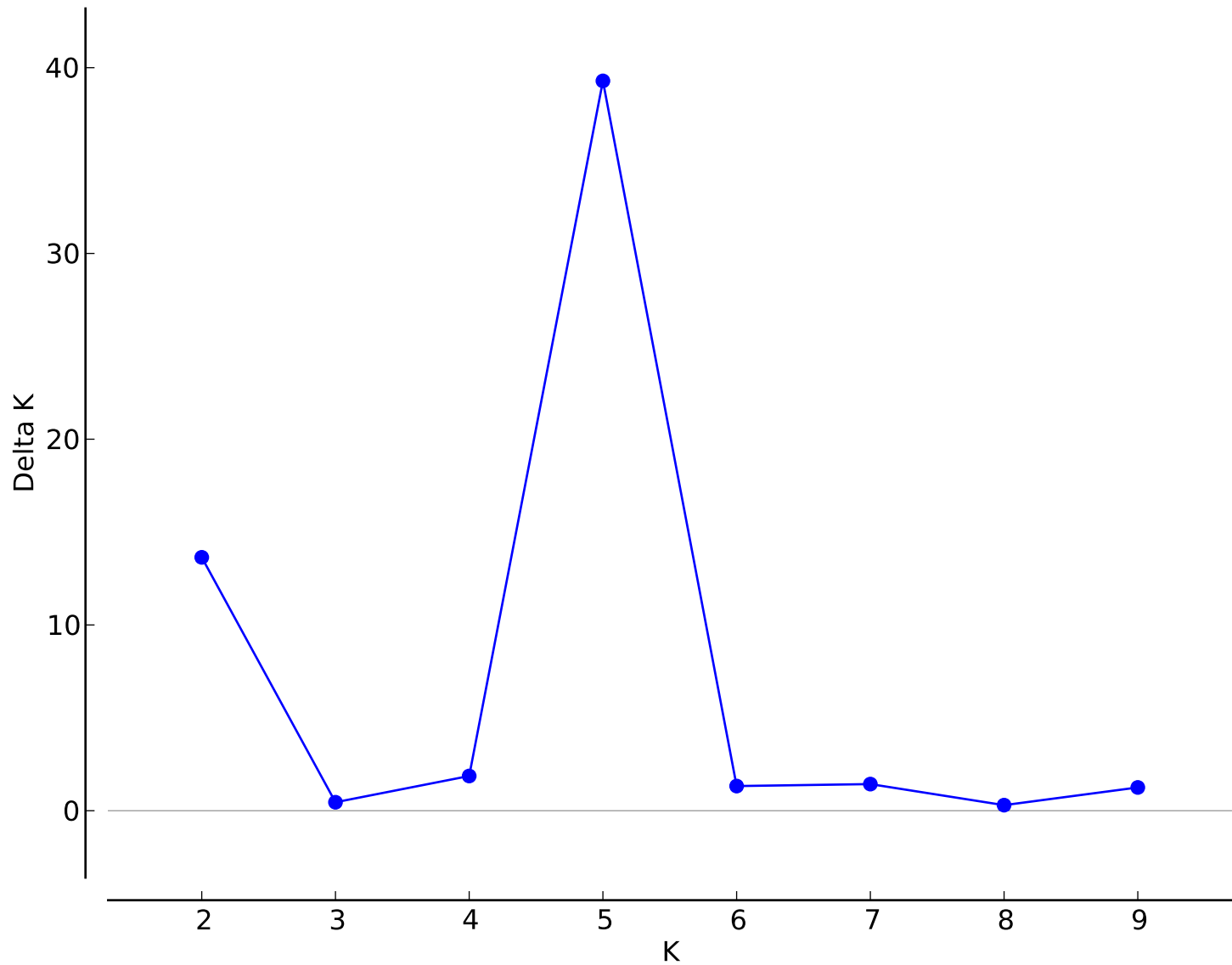

Supplement: Supplementary file 2 [file ECE3-7-9234-s002.pdf]

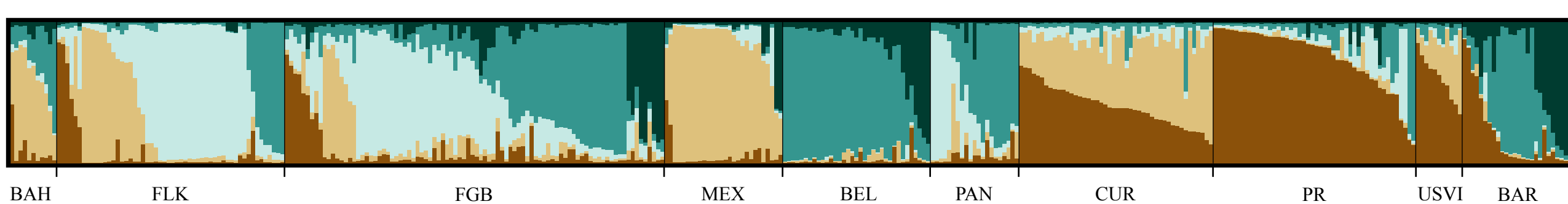

Supplement: Supplementary file 3 [file ECE3-7-9234-s003.pdf]
