# Supplementary material for: Population structure and connectivity of the mountainous star coral, Orbicella faveolata, throughout the wider Caribbean region
Source: Ecol Evol. 2017 Oct 3;7(22):9234–46. doi: 10.1002/ece3.3448 (PMC5696396; doi:10.1002/ece3.3448)
Supplement: Supplementary file 9 [file ECE3-7-9234-s009.docx]

**SUPPORTING INFORMATION**

**Appendix S1**

*Pooling populations within sampling sites*

The primary interest of this investigation was in large-scale patterns of population structure across the wider Caribbean, and therefore we pooled all sampling locations from each reef system into a single population to avoid overcomplicating regional patterns. For the samples collected from immediately adjacent reefs (i.e., two in Puerto Rico and two in USVI), this is unlikely to create a problem. However, within certain reef systems, the sampling locations were considerably far apart (i.e., Florida Keys, Curaçao and FGB).

In regard to the Florida Keys, only six samples were collected from each of the upper Keys sites, while 49 were collected from the Lower Keys site. We were not comfortable assuming that six samples would accurately represent the true allele frequencies at those locations, so we elected to group all the Florida Keys samples into a single population. Similarly, the 46 samples from Curaçao were almost evenly split between the three collection sites, leaving 14-16 samples per site. Again, rather than base allele frequency distributions on small neighboring populations, we pooled these sites into a larger population representing the entire Curaçao reef system.

The two populations from the Flower Garden Banks, however, had sufficiently large sample sizes (n = 39, 51) and are uniquely separated by 13 miles of open ocean (60-150 m depth). Thus, we ran analyses both with and without pooling the sites and found that the populations on the banks were quite similar genetically. The pairwise F_ST_ between the two populations was 0.012 and non-significant after Bonferroni correction, which was the lowest value for any pairwise comparison in that analysis. Due to the geographic proximity of the two sites, the genetic similarity warranted pooling them into a larger population for the basin-wide study.

*Analysis of Null Alleles*

In developing the microsatellites used in this study, Davies *et al.* (2013b) found evidence for null alleles at three of the four loci associated with substantial heterozygote deficit, suggesting that the observed pattern may be due to the presence of null alleles. Re-analysis with the expanded dataset of this study corroborates this finding, in that the highest frequencies of null alleles estimated using the EM algorithm of Dempster *et al.* (1977) were found in the same four loci. It is important to note, however, that all estimates were relatively low (max $\hat{r}$ = 0.22; Table S2). Chapuis and Estoup (2007) demonstrate through simulations that a null allele rate of approximately 20% has a negligible effect on estimates of population structure.

To assess the effect of null alleles on our analyses, pairwise F_ST_ values between sites were adjusted using the excluding null allele (ENA) method executed in FreeNA, as recommended by Chapuis and Estoup (2007). A Mantel test between uncorrected and ENA-corrected pairwise F_ST_ (9999 permutations) showed these values to be very similar and strongly correlated (mean difference: 0.0001, r = 0.985, p-value = 0.0001; Figure S1), indicating that the presence of null alleles does not significantly alter downstream analyses. For this reason, and due to the relatively low frequency of null alleles, we elected to retain all nine microsatellite loci in our analysis.

**Table S1. Single locus estimates of heterozygote deficiency.** *N*: number of unique multilocus genets, *A_e_*: effective number of alleles, *HWE*: *P* value of Fishers’ exact test for heterozygote deficit based on 10,000 dememorization steps with 500 batches of 10,000 random permutations, *F_IS_*: inbreeding coefficient. All values shaded in grey are significant at the α = 0.05 level following sequential Bonferroni correction. Site abbreviations are as in Table 1.

**Table S2. Single locus estimates of null allele frequency based on EM algorithm of Dempster *et al.* (1977)**. Site abbreviations are as in Table 1.

**Table S3. Tests of linkage disequilibrium between pairs of loci.** *P* values for significance tests are based on 7200 random permutations. All values shaded in grey are significant at the α = 0.05 level following sequential Bonferroni correction. Site abbreviations are as in Table 1.

**Table S4. Number and proportion of clonal genotypes and individuals**. *N*: total number of sampled colonies. Site abbreviations are as in Table 1.

**Table S5. Pairwise estimates of** $\mathbf{G}_{\mathbf{ST}}^{\mathbf{''}}$ **between all populations**. All values shaded in grey are significant at the α = 0.05 level following sequential Bonferroni correction. Site abbreviations are as in Table 1.

**Figure S1. Correlation between uncorrected pairwise estimates of F_ST_ and pairwise estimates corrected by excluding null alleles**.

**Figure S2. Estimation of the optimum number of genetic clusters (K) from STRUCTURE analysis based on the ad hoc statistic, ΔK (Evanno *et al.* 2005)**.

ΔK = mean( | L”(K) | ) / sd(L(K)).

**Figure S3. STRUCTURE population assignments displayed side-by-side.** As in Figure 4, thin vertical columns in each population block represent individual samples and their associated probability of assignment to K = 5 color-coded genetic clusters (Brown, Tan, Light Blue, Teal, and Dark Blue). Site abbreviations are as in Table 1 and are organized along the axes by relative geographic location in the basin.
